# Investigation of the interaction between Genetic Risk Score (GRS) and fatty acid quality indices on mental health among overweight and obese women

**DOI:** 10.1186/s12905-023-02491-0

**Published:** 2023-08-04

**Authors:** Niloufar Rasaei, Mahsa Samadi, Alireza Khadem, Seyedeh Fatemeh Fatemi, Fatemeh Gholami, Khadijeh Mirzaei

**Affiliations:** 1 Department of Community Nutrition, School of Nutritional Sciences and Dietetics, Tehran University of Medical Sciences (TUMS), Tehran, Iran; 2 Network of Interdisciplinarity in Neonates and Infants (NINI), Universal Scientific Education and Research Network (USERN), Tehran, Iran; 3 Department of Nutrition, Science and Research Branch, Islamic Azad University, Tehran, Iran; 4 Department of Nutrition, Faculty of Medicine, Mashhad University of Medical Sciences, Mashhad, Iran; 5 Food Microbiology Research Center, Tehran University of Medical Sciences, Tehran, Iran

**Keywords:** Genetic risk score, Dietary fat quality indices, Mental health, Overweight, Obese

## Abstract

**Background & aims:**

Mental disorders are associated with dietary fatty acids and genome-wide association studies have found multiple risk loci robustly related to depression, anxiety, and stress. The aim of this study is to investigate the interaction of genetic risk score (GRS) and dietary fat quality indices on mental health.

**Methods:**

This cross-sectional study included 279 overweight and obese women for N6/N3 ratio and 378 overweight and obese women for CSI aged 18–68 years. Using reliable and verified standard protocols, body composition, anthropometric indices, blood pressure, physical activity, and dietary fat quality were measured. Serum samples were used to determine biochemical tests. A genetic risk score (GRS) was calculated using the risk alleles of the three SNPs. A generalized linear model (GLM) was applied to assess the interactions between GRS and fat quality indices. Mental health was evaluated using Depression Anxiety Stress Scales (DASS-21).

**Results:**

The mean (± SD) age and BMI of our participants were 36.48 (8.45) and 30.73 (3.72) kg/m2 respectively. There was a marginally significant mean difference among tertiles of the CSI in terms of stress (P = 0.051), DASS-21 (P = 0.078) in the crude model. After adjusting for age, energy intake, physical activity and BMI in model 1, there was a positive interaction between GRS and T3 of N6/N3 ratio on anxiety (β = 0.91, CI = 0.08,1.75, P = 0.031), depression (β = 1.05, CI = 0.06,2.04, P = 0.037), DASS-21 (β = 2.22, CI= -0.31,4.75, P = 0.086).

**Conclusion:**

Our findings indicate that higher ratio of N-6 to N-3 considering genetics were predictive of mental disorder in our population.

## Introduction

Depression and anxiety are the most frequent psychiatric disorders [1], which are significantly characterized by psychological symptoms including decreased mental activity, mood changes, cognitive impairment, phobia, fatigue, and heart palpitations [2, 3]. The global worldwide prevalence of depression, anxiety, and stress has been reported 60.8%, 73%, and 62.4%, respectively. According to a population-based studies in Iran, the prevalence of depression and anxiety has been estimated 4.1% and 15.6%, respectively [4–6]. Among the various factors related to mental disorders, genetic variations and dietary intake play an important role [7, 8].

Lipids are involved in the function of neurons in the brain, which lead to depression and anxiety-related behaviors by reducing membrane fluidity [9]. A new dietary fat quality index as Cholesterol-Saturated Fat Index (CSI) was proposed by Connor et al. during the previous decade [10]. This index is known as a dietary self-monitoring tool, which indicates the content of dietary cholesterol and saturated fat. A lower CSI is related to lower saturated fat and cholesterol, and low atherogenicity [10]. Alongside CSI, the omega-6/omega-3 essential fatty acids (EFA) ratio was introduced by Simopoulos et al. The N6/N3 ratio measures the balance between omega-6 and omega-3 polyunsaturated fatty acids in the diet, which are important for brain function and inflammation regulation. In general, a higher N6/N3 ratio indicates a higher intake of omega-6 fatty acids than omega-3 fatty acids, while a lower ratio indicates a higher intake of omega-3 fatty acids [11]. Several studies evaluated the effects of dietary fatty acids on mental disorders and found controversial results. Previous studies have shown that daily intakes of EPA and DHA supplementation have an effective role in decreasing depressive symptoms in patients with anxiety, major depressive disorder, and stress [12–14]. On the other hand, the results of a meta-analysis of 31 trials indicated that long-chain omega-3 fatty acids consumption may have no significant effect on preventing symptoms of mental disorders such as depression or anxiety [15]. Based on another review of 34 studies, there is currently insufficient and reliable evidence of the effect of omega-3 fatty acids in the prevention and treatment of the depressive disorders [16]. Studies have proven that saturated fatty acids (SFA) negatively affect membrane fluidity and brain functions [17]. A positive association was indicated between SFAs and increased symptoms of depression and anxiety disorders [18, 19]. Of note is that, genetic factors have been known as a significant risk factor for mental disorders [8]. Developments in genome-wide association studies (GWAS) have provided the investigation of genetic risk scores (GRS),which are considered for each single nucleotide polymorphism (SNP) with the collection of risk alleles [20]. Sum of 30 depression-related SNPs were contained in a GRS to investigate and predict depression disorder risk in one study [21]. Here, three new genes of Cryptochrome (CRY), Melanocortin-4 Receptor (MC4R), and Caveolin (CAV) were associated with mental disorders-related SNPs in GWAS [22–24]. Other authors found significant associations between GRS with major depression [20, 25], anxiety [26], and stress [27]. Regarding the “gene-environment interaction” hypothesis [28], dietary fatty acids are related to GRS [29]. Based on the high prevalence rates of depression and anxiety globally and in Iran and its impact on individuals and society, there remains a critical gap in understanding the complex interplay between genetic factors and dietary intake in the context of mental health. This gap needs to be addressed in order to uncover novel insight into the underlying mechanisms of mental disorders and to develop targeted interventions and preventative measures. However, to the best of our knowledge, there is no literature on the interaction between GRS and dietary fat quality index on mental disorders. Therefore, the aim of this study is to investigate the interaction between GRS including MC4R (rs17782313), CAV-1 (rs3807992), and Cry-1 (rs2287161) with dietary fat quality indices based on CSI and omega-6/omega-3 EFA ratio in relation to mental health, particularly among overweight and obese individuals.

## Materials and methods

### Study population

This cross-sectional study included 279 overweight and obese women for N6/N3 ratio and 378 overweight and obese women for CSI who were referred to health centers of Tehran University of Medical Science. The inclusion criteria were as follows: body mass index (BMI) of 25 to 40 kg/m^2^ and in the 18–68 age range. The exclusion criteria were as follows: history of cardiovascular or thyroid disease, malignancies, hepatic or renal disease, all types of diabetes, acute or chronic diseases, presence of menopause, pregnancy or lactation, following a specific diet or supplement usage to lose weight over the past year, glucose and lipid lowering drugs, medicine consumption for hypertension, and smoking. Written informed consent was obtained from all subjects. The protocol of the study was approved by the ethics committee of the TUMS (Ethics number: IR.TUMS.VCR.REC.1399.636). All methods were performed in accordance with relevant guidelines/regulations.

### Anthropometric and blood pressure measurements

Bioelectrical impedance analyzer (BIA) (InBody 770 scanner from InBody Co. (Seoul, Korea)) was used to analyze body composition and weight, body fat mass, fat-free mass, body fat percent, and visceral fat of the participants by following the manufacturer’s protocol [30]. Participants were asked to remove metal objects such as rings, earrings, and watches as well as coats, sweaters, and shoes. Moreover, the height was measured using a non-elastic tape, to the nearest to 0.5 cm, in a standing position and unshod. For measuring the BMI, the weight (in kilograms) was divided into height squared (in square meters). According to World Health Organization, overweight was defined BMI 25-29.9 kg/m^2^ and obesity grades 1, 2, and 3 were defined as BMI 30-34.9 kg/m^2^, BMI 35-39.9, and BMI 40 kg/m^2^, respectively. Waist circumference (WC) and hip circumference, respectively, were measured, after expiration, in the narrowest area of the torso and maximum posterior extension of the buttocks, with accuracy of 0.5 cm. The waist-to-hip ratio (WHR) was calculated by dividing the waist circumference (cm) by hip circumference (cm). Blood pressure (BP) was measured for two times after 5 min rest, using an appropriate cuff. Averaged value of the two readings was recorded.

### Physical activity assessment

Participant’s physical activity (PA) was appraised using a reliable and validated international physical activity questionnaire-short form (IPAQ) through a face-to face interview. The metabolic equivalent hours per week (METs-h / week) was measured for each individuals in the last week [31].

### Biochemical and hormonal determination

Serum samples were collected after overnight fasting (10–12 h) at the Nutrition and Biochemistry Laboratory of the School of Nutritional and Dietetics, TUMS. All samples were centrifuged, stored at − 80 °C, and analyzed via a single assay technique. We measured fasting blood glucose (FBS) using glucose oxidase-phenol 4-aminoantipyrine peroxidase (GOD-PAP) and Triglyceride (TG) was assayed using glycerol-3-phosphate oxidase–phenol 4-aminoantipyrine peroxidase (GPOPAP) enzymatic endpoint. Direct enzymatic clearance assay was used to measure high-density lipoprotein (HDL), low-density lipoprotein (LDL), and total cholesterol. Standard protocols were applied to measure insulin and hs-CRP. The homeostatic model assessment insulin resistance (HOMA-IR) was used to assess insulin resistance (mIU/ml), with the following formula: [fasting plasma glucose (mmol/l) × insulin (IU/l)] / 22.5) [32]. All evaluations were performed using Randox Laboratories (Hitachi 902) kits.

### Mental health assessment

Mental health status of participants was determined using the 21-item self-report version of the Depression Anxiety Stress Scales (DASS-21), a valid tool that provides states of depression, anxiety, and stress [33]. To identify the final score, DASS-21 scores were multiplied by two and divided in to three categories as cutoff points for having depression (≥ 10), anxiety (≥ 8), and stress (≥ 15).

### Dietary intake assessment

We used a 147-item validated semi-quantitative standard food frequency questionnaire (FFQ) to evaluate dietary intakes of all participants over the preceding year [34]. This questionnaire was completed by an expert dietician and subjects reported the frequency of each food item on a daily, weekly, monthly, or yearly basis. The extracted FFQ values were then entered into an excel program which determined the weight (grams) of each food item. Total energy, macronutrients, and micronutrients were analyzed using the NUTRITIONIST 4 (First Data Bank, San Bruno, CA) food analyzer [35].

### Dietary fat quality indices

Both cholesterol-saturated fat index (CSI) and the ratio of omega-6/omega-3 (N-6/N-3) essential fatty acids are considered dietary fat quality indices which are calculated by following formulas:

- CSI: represents the cholesterol and saturated fat content of food, which helps a person in self-care against high levels of cholesterol [36].

CSI = Cholesterol / Saturated fat

- The N-6 to N-3 ratio: Omega 6 and Omega 3 are two essential fats, categorized as PUFAs [37, 38].

N-6 to N-3 ratio: total Omega 6/ total Omega

### Genotyping and GRS

DNA was extracted from whole blood samples by using salting out methods [39]. The integrity and concentration of extracted DNA were assessed using 1% agarose gel and the Nanodrop 8000 Spectrophotometer (Thermo Scientific, Waltham, MA, USA), respectively. Single nucleotide polymorphisms (SNPs) were genotyped using the TaqMan Open Array (Life Technologies Corporation, Carlsbad, CA, USA) [40]. The forward primer of CAV-1 (rs3807992) is 3′AGTATTGACCTGATTTGCCATG 5′ and the reverse primer is 5′ GTCTTCTGGAAAAAGCACATGA 3′. The fragments containing three genotypes including GG, AA, and GA were distinguished. The forward and reverse primers of Cry1 (rs2287161) are 5′-GGAACAGTGATTGGCTCTATCT − 3′ and 5′-GGTCCTCGGTCTCAAGAAG-3′, respectively. Pieces containing three genotypes were distinguished: CC, GG, and GC. Based on a previous study, the MC4R gene primer was determined [41]. The sequence of MC4R (rs17782313) primers used are as follows: primers forward: 5- AAGTTCTACCTACCATGTTCTTGG-3; reverse: 5-.

TTCCCCCTGAAGCTTTTCTTGTCATTTTGAT-3. Then, Fragments concluding three genotypes were detected: CC, TT, and CT. Finally, we computed the GRS by summing up three SNPs [CAV-1 (rs3807992), Cry-1 (rs2287161), and MC4R (rs17782313)] that, based on GWAS and other studies, had been linked to obesity-related traits [42–44]. Each SNP was coded as 0, 1, or 2 according to the risk alleles for higher BMI. The unweighted GRS was determined using the risk alleles of the three SNPs. The GRS ranges from 0 to 6 and higher scores reflect greater genetic predisposition to high BMI on the GRS scale [45].

### Statistical analyses

All data were analyzed by SPSS version 23.0 (SPSS, Chicago, IL, USA), and a P-value < 0.05 was considered statistically significant and interaction P-value < 0.1 was set as mariginally significant. The Kolmogorov-Smirnov test was utilized to find out the normality distribution of data. Demographic characteristics of participants were reported by mean ± standard deviation, minimum and maximum. A one-way analysis of variance (ANOVA) test was used to compare anthropometric indices, FBS, BP, and lipid profile between participants. Analysis of covariance (ANCOVA) was applied to remove confounding results. A generalized linear model (GLM) was used in both crude and adjusted models to assess the interactions between GRS and fatty acid quality indices. The results were adjusted for energy intake, age, BMI, and PA.

## Results

### Study population characteristics

A total of 279 overweight and obese women for N6/N3 ratio and 378 overweight and obese women for CSI aged enrolled in this cross-sectional study. The mean (± SD) age, height, weight, and BMI of partici pants were 36.48 (8.45), 161.32 (5.82) cm, 79.99 (10.88) kg, 30.73 (3.72) kg/m^2^, respectively. Also, the mean (± SD) of biochemical variables including FBS, TG, HDL, LDL, TC, and hs-CRP of participants was 87.25 (9.66), 121.12 (69.84), 46.68 (10.61), 94.25 (23.84), 183.71 (35.80), and 4.77 (4.69), respectively. In terms of job and education, most participants were housekeeper 164 (58.8%), bachelor’s degree and higher 132 (47.3%), respectively.

### General characteristics of study population according to tertiles of CSI and N6/N3

The baseline characteristics of study participants, categorized according to tertiles of CSI and N6/N3 and GRS, were presented in Table 1. As shown in this table, in the crude model, there was a significant mean difference among tertiles of the CSI in terms of age (P = 0.018), and the significant mean difference in terms of FFM (P = 0.009) and marginally significant for PA (P = 0.082), height (P = 0.058), WHR (P = 0.075) were observed among tertiles of the N6/N3. After adjustment with confounders, including age, BMI, physical activity, and energy intake, the education (P = 0.009) of participants among tertiles of the CSI became significant, and there was a significant mean difference in terms of HOMA index (P = 0.033) and the marginally significant for FBS (P = 0.055) among tertiles of the N6/N3.

**Table 1:** General characteristics of study population according to tertiles of CSI (378) and N6/N3 (279) in obese and overweight women

**Variables†**	**CSI**					
	**Mean ± SD**				**P-value**	**P-value**_**b**_
	**T**_**1**_ **(n = 126)**	**T**_**2**_ **(n = 126)**		**T**_**3**_ **(n = 126)**		
Age (years)	37.87 ± 8.95	37.34 ± 9.22		34.81 ± 9.19	**0.018**	0.473
PA (MET-min/week)	839.32 ± 1027.52	1092.78 ± 1205.06		1048.35 ± 1046.47	0.181	0.366
**Anthropometric measurements**						
Weight (kg)	79.79 ± 10.58	80.12 ± 11.20		81.95 ± 12.10	0.265	0.868
Height (cm)	160.68 ± 5.86	161.27 ± 5.86		161.64 ± 5.63	0.417	0.963
WC (cm)	98.62 ± 9.09	98.73 ± 9.52		100.19 ± 10.09	0.348	0.841
WHR	0.93 ± 0.04	0.93 ± 0.05		0.93 ± 0.05	0.555	0.787
BMI (kg/m^2^)	30.95 ± 3.70	30.76 ± 3.77		31.33 ± 4.07	0.495	0.553
BF (%)	42.03 ± 5.27	41.60 ± 4.95		42.48 ± 5.80	0.427	0.628
VFA (cm^2^)	163.06 ± 37.75	163.45 ± 36.85		170.32 ± 37.99	0.226	0.514
FFM (kg)	46.06 ± 5.34	46.48 ± 5.48		46.53 ± 5.77	0.758	0.558
BFM (kg)	33.88 ± 7.79	33.59 ± 7.60		35.32 ± 8.55	0.184	0.589
**Blood pressure**						
SBP (mmHg)	112.88 ± 13.58	111.01 ± 14.02		109.54 ± 12.71	0.280	0.708
DBP (mmHg)	78.52 ± 9.84	77.66 ± 8.49		76.24 ± 10.63	0.314	0.646
**Biochemical variables**						
FBS (mg/dl)	87.98 ± 10.60	87.14 ± 9.70		86.41 ± 8.23	0.621	0.992
TC (mg/dl)	184.75 ± 31.24	184.22 ± 39.71		181.56 ± 36.17	0.857	0.661
TG (mg/dl)	123.69 ± 81.12	124.06 ± 70.69		113.43 ± 49.64	0.602	0.701
HDL (mg/dl)	47.40 ± 10.41	46.42 ± 12.13		46.09 ± 8.42	0.732	0.874
LDL (mg/dl)	95.08 ± 23.11	92.84 ± 25.15		95.16 ± 23.15	0.779	0.308
Insulin (mIU/mL)	1.19 ± 0.23	1.22 ± 0.21		1.22 ± 0.23	0.684	0.580
HOMA index	3.42 ± 1.40	3.17 ± 1.17		3.48 ± 1.27	0.269	0.422
hs.CRP (mg/l)	4.58 ± 4.28	5.05 ± 4.75		5.56 ± 4.63	0.230	0.265
**Education%(n)**					0.166	**0.009**
Illiterate	75.0 (3)	25.0 (1)		0.0 (0)		
Primary education	46.2 (6)	30.8 (4)		23.1 (3)		
Intermediate Education	52.0 (13)	24.0 (6)		24.0 (6)		
High school education	50.0 (4)	12.5 (1)		37.5 (3)		
Diploma	30.8 (36)	38.5 (45)		30.8 (36)		
Postgraduate education	44.4 (12)	33.3 (9)		22.2 (6)		
Bachelor’s degree and higher	28.0 (51)	32.4 (59)		39.6 (72)		
**Job%(n)**					0.575	0.529
Housekeeper	33.6 (73)	34.1 (74)		32.3 (70)		
Labor	25.0 (1)	75.0 (3)		0.0 (0)		
Management employee	35.4 (23)	32.3 (21)		32.3 (21)		
Non-managerial employee	30.8 (16)	30.8 (16)		38.5 (20)		
household jobs	50.0 (8)	12.5 (2)		37.5 (6)		
University student	22.2 (4)	44.4 (8)		33.3 (6)		
**Marriage%(n)**					0.257	0.275
Married	34.8 (94)	34.8 (94)		30.4 (82)		
Single	30.0 (27)	28.9 (26)		41.1 (37)		
Away from spouse more than 6 month	50.0 (1)	50.0 (1)		0.0 (0)		
Dead spouse	0.0 (0)	0.0 (0)		100.0 (3)		
Divorce	27.3 (3)	36.4 (4)		36.4 (4)		
**Supplementation%(n)**					0.138	0.142
Yes	30.3 (47)	36.8 (57)		32.9 (51)		
No	38.2 (65)	27.1 (46)		34.7 (59)		
**Variables**†	**N6/N3**					
	**Mean ± SD**				**P-value**	**P-value**_**b**_
	**T**_**1**_ **(n = 93)**		**T**_**2**_ **(n = 93)**	**T**_**3**_ **(n = 93)**		
Age (years)	35.95 ± 8.20		36.08 ± 8.45	37.40 ± 8.72	0.434	0.294
PA (MET-min/week)	960.36 ± 926.07		1192.29 ± 1445.85	812.75 ± 727.60	**0.082**	0.148
**Anthropometric measurements**						
Weight (kg)	81.12 ± 10.74		80.84 ± 11.89	78.01 ± 9.77	0.098	0.788
Height (cm)	162.02 ± 5.47		161.79 ± 5.77	160.15 ± 6.09	**0.058**	0.655
WC (cm)	98.81 ± 9.13		99.62 ± 10.11	96.79 ± 8.49	0.103	0.238
WHR	0.92 ± 0.04		0.94 ± 0.05	0.92 ± 0.04	**0.075**	0.263
BMI (kg/)	30.90 ± 3.93		30.91 ± 3.63	30.37 ± 3.61	0.532	0.462
BF (%)	41.20 ± 5.88		41.05 ± 5.15	41.55 ± 4.91	0.809	0.464
VFA (cm^2)^	162.88 ± 37.06		161.11 ± 40.92	158.08 ± 33.64	0.677	0.766
FFM (kg)	47.07 ± 4.96		47.48 ± 5.84	45.22 ± 5.07	**0.009**	0.189
BFM (kg)	33.99 ± 7.88		33.73 ± 7.99	32.42 ± 6.86	0.324	0.550
**Blood pressure**						
SBP (mmHg)	110.35 ± 14.18		112.51 ± 12.88	110.59 ± 13.55	0.503	0.294
DBP (mmHg)	76.94 ± 10.37		78.08 ± 9.42	77.62 ± 9.10	0.727	0.270
**Metabolic factors**						
FBS (mg/dl)	87.06 ± 9.31		86.35 ± 9.12	88.22 ± 10.43	0.468	**0.055**
TC (mg/dl)	178.53 ± 29.15		184.55 ± 37.64	187.71 ± 39.35	0.260	0.103
TG (mg/dl)	118.33 ± 67.37		121.08 ± 72.83	123.72 ± 70.10	0.888	0.240
HDL (mg/dl)	46.18 ± 10.16		47.51 ± 11.04	46.42 ± 10.70	0.716	0.810
LDL (mg/dl)	92.81 ± 20.49		94.91 ± 24.87	94.98 ± 25.90	0.813	0.853
Insulin (mIU/mL)	1.21 ± 0.24		1.23 ± 0.22	1.19 ± 0.21	0.537	0.324
HOMA index	3.22 ± 1.27		3.23 ± 1.27	3.54 ± 1.30	0.198	**0.033**
hs.CRP (mg/l)	4.83 ± 4.48		5.12 ± 5.02	4.36 ± 4.57	0.543	0.373
**Education%(n)**					0.608	0.462
Illiterate	0.0 (0)	66.7 (2)		33.3 (1)		
Primary education	30.8 (4)	53.8 (7)		15.4 (2)		
Intermediate Education	35.3 (6)	23.5 (4)		41.2 (7)		
High school education	28.6 (2)	42.9 (3)		28.6 (2)		
Diploma	37.0 (30)	32.1 (26)		30.9 (25)		
Postgraduate education	16.7 (4)	41.7 (10)		41.7 (10)		
Bachelor’s degree and higher	34.8 (46)	31.1 (41)		34.1 (45)		
**Job%(n)**					0.291	0.215
Housekeeper	35.4 (58)	34.8 (57)		29.9 (49)		
Labor	33.3 (1)	66.7 (2)		0.0 (0)		
Management employee	19.6 (9)	34.8 (16)		45.7 (21)		
Non-managerial employee	38.9 (14)	22.2 (8)		38.9 (14)		
household jobs	16.7 (1)	33.3 (2)		50.0 (3)		
University student	38.9 (7)	33.3 (6)		27.8 (5)		
**Marriage%(n)**					0.575	0.614
Married	32.6 (70)	34.0 (73)		33.5 (72)		
Single	33.3 (18)	33.3 (18)		33.3 (18)		
Away from spouse more than 6 month	0.0 (0)	0.0 (0)		100.0 (1)		
Dead spouse	100.0 (2)	0.0 (0)		0.0 (0)		
Divorce	40.0 (2)	40.0 (2)		20.0 (1)		
**Supplementation%(n)**					0.371	0.249
Yes	35.1 (46)	36.6 (48)		28.2 (37)		
No	32.6 (31)	30.5 (29)		36.8 (35)		

BF%; body fat percentage; BFM: body fat mass; BMI: body mass index; CSI: cholestrol to saturated fat index; DBP: diastolic blood pressure; FBS: fasting blood sugar; FFM: fat free mass;: HDL: high density lipoprotein; HOMA; homeostatic model assessment; hs-CRP: high-sensitivity C-reactive protein; PA: physical activity; SD: standard deviation; SBP: systolic blood pressure; T: tertile; TC: total cholesterol; TG: triglyceride; VFA: visceral fat area; WC: waist circumference

† Calculated by analysis of variance (ANOVA)

b: Adjusted for age, BMI, physical activity, and total energy intake

a: BMI consider as a collinear variable for anthropometric measurements and these variables adjusted for Age, physical activity, and total energy intake

p < 0.05 was considered significant

### Dietary intake of study population according to tertiles of CSI and N6/N3

Dietary intakes of participants among tertiles of CSI and N6/N3 ratio were presented in Table 2. After adjustment with the energy intake, mean differences of refined grains, high fat dairy, low-fat dairy, fish, poultry, egg, fast food, red meat, protein, carbohydrate, total cholesterol, polyunsaturated fatty acid (PUFA), saturated fatty acid (SFA), linoleic acid, eicosapentaenoic acid (EPA), docosahexaenoic acid (DHA) (P = 0.001), vegetables (P = 0.003), sweet desert (P = 0.044), total fat (P = 0.006), oleic acid (P = 0.009) were significant across tertiles of CSI, also a significant mean difference was observed among tertiles of the N6/N3 in terms of fruits (P = 0.046), sweet desert (P = 0.035), monounsaturated fatty acid (MUFA) (P = 0.034), PUFA (P = 0.029), oleic acid (P = 0.027), linoleic acid (P = 0.030).

**Table 2:** Dietary intake of study population according to tertiles of CSI (378) and N6/N3 (279) in obese and overweight women

**Variables†**	**CSI**			
	**Mean ± SD**			**p-value**
	**T**_**1**_ **(n = 126)**	**T**_**2**_ **(n = 126)**	**T**_**3**_ **(n = 126)**	
**Food group**				
Whole grains (g/d)	60.65 ± 59.95	64.81 ± 57.51	94.32 ± 101.21	0.346
Refined grains (g/d)	329.03 ± 210.11	375.64 ± 188.06	386.16 ± 207.17	**0.001**
Nuts (g/d)	10.15 ± 12.26	15.12 ± 20.69	20.78 ± 21.41	0.741
Legumes (g/d)	40.35 ± 33.72	49.66 ± 42.61	45.80 ± 40.44	0.212
Vegetables (g/d)	282.37 ± 190.02	410.58 ± 251.52	433.96 ± 245.43	**0.003**
Fruits (g/d)	359.96 ± 288.70	471.84 ± 323.43	518.56 ± 321.90	0.355
High fat dairy (ml/d)	49.50 ± 71.59	76.93 ± 116.12	150.43 ± 189.28	**0.001**
Low-fat dairy (ml/d)	200.60 ± 136.87	310.65 ± 224.68	367.16 ± 249.14	**0.001**
Fish (g/d)	7.06 ± 6.41	11.85 ± 11.74	14.10 ± 15.56	**0.001**
Poultry (g/d)	21.89 ± 17.23	33.70 ± 25.36	52.21 ± 54.94	**0.001**
Egg (g/d)	12.59 ± 7.10	22.17 ± 9.49	38.60 ± 22.42	**0.001**
Fast food (g/d)	14.48 ± 16.92	15.50 ± 18.41	32.47 ± 37.43	**0.001**
Red meat (g/d)	12.09 ± 8.28	20.95 ± 15.96	32.14 ± 27.39	**0.001**
Sweet desert (g/d)	68.36 ± 82.18	78.25 ± 74.58	93.30 ± 75.11	**0.044**
**Nutrient intake**				
Energy (kcal/d)	2141.38 ± 670.84	2592.78 ± 696.85	3143.79 ± 725.64	-
Protein (g/d)	67.48 ± 19.00	90.07 ± 21.73	116.48 ± 31.16	**0.001**
Carbohydrate (g/d)	307.93 ± 108.21	377.07 ± 121.67	429.97 ± 111.97	**0.001**
Total fat (g/d)	78.00 ± 31.69	89.47 ± 26.98	116.52 ± 33.07	**0.006**
TC (g/d)	156.81 ± 29.70	241.09 ± 25.05	395.06 ± 91.37	**0.001**
MUFA (g/d)	27.06 ± 11.69	29.95 ± 8.87	37.72 ± 10.50	0.052
PUFA (g/d)	18.36 ± 10.74	18.92 ± 7.51	22.46 ± 8.37	**0.001**
SFA (mg/d)	20.83 ± 6.96	26.14 ± 6.58	38.07 ± 12.44	**0.001**
Trans fat	0.001 ± 0.001	0.001 ± 0.001	0.001 ± 0.003	0.163
Oleic acid (g/d)	24.55 ± 12.45	26.42 ± 8.79	34.78 ± 11.19	**0.009**
Linolenic acid (g/d)	1.01 ± 0.67	1.15 ± 0.53	1.44 ± 0.62	0.628
Linoleic acid (g/d)	16.32 ± 10.18	16.35 ± 7.20	19.06 ± 7.87	**0.001**
EPA (g/d)	0.01 ± 0.02	0.03 ± 0.03	0.03 ± 0.04	**0.001**
DHA (g/d)	0.06 ± 0.06	0.10 ± 0.11	0.13 ± 0.14	**0.001**
**Variables**†	**N6/N3**			
	**Mean ± SD**			**P-value** ^*^
	**T1(n = 93)**	**T2(n = 93)**	**T3(n = 93)**	
**Food group**				
Whole grains (g/d)	76.88 ± 67.78	70.52 ± 59.97	41.42 ± 38.36	0.175
Refined grains (g/d)	489.62 ± 239.45	340.17 ± 194.30	272.29 ± 117.29	0.462
Nuts (g/d)	21.11 ± 19.00	15.81 ± 17.75	6.95 ± 6.07	0.369
Legumes (g/d)	51.82 ± 40.69	52.32 ± 44.80	36.50 ± 31.08	0.189
Vegetables (g/d)	439.80 ± 243.54	417.86 ± 256.50	289.23 ± 183.76	0.060
Fruits (g/d)	750.11 ± 382.63	439.53 ± 243.73	325.48 ± 209.00	**0.046**
High fat dairy (ml/d)	142.61 ± 178.93	85.27 ± 120.21	34.16 ± 55.29	0.290
Low-fat dairy (ml/d)	371.77 ± 281.11	299.61 ± 205.06	232.10 ± 152.00	0.818
Fish (g/d)	13.75 ± 15.65	11.24 ± 11.07	9.36 ± 8.81	0.996
Poultry (g/d)	45.60 ± 55.96	31.70 ± 29.99	28.12 ± 23.10	0.328
Egg (g/d)	25.27 ± 17.06	22.53 ± 13.63	17.38 ± 10.67	0.385
Fast food (g/d)	27.02 ± 34.16	17.00 ± 20.52	13.62 ± 16.33	0.710
Red meat (g/d)	31.64 ± 20.16	20.75 ± 19.16	12.47 ± 8.39	0.058
Sweet desert (g/d)	110.81 ± 83.91	69.46 ± 62.22	45.47 ± 55.59	**0.035**
**Nutrient intake**				
Energy (kcal/d)	3468.72 ± 402.67	2545.78 ± 190.36	1799.81 ± 271.01	
Protein (g/d)	114.98 ± 24.09	87.51 ± 17.49	62.37 ± 13.30	0.584
Carbohydrate (g/d)	502.95 ± 82.83	353.96 ± 47.13	255.92 ± 53.31	0.099
Total fat (g/d)	122.50 ± 27.88	95.28 ± 20.53	63.74 ± 15.19	0.096
Total cholesterol (g/d)	326.34 ± 123.41	241.50 ± 75.48	189.51 ± 56.43	0.438
MUFA (g/d)	39.10 ± 9.87	32.22 ± 9.23	21.80 ± 6.55	**0.034**
PUFA (g/d)	24.25 ± 7.54	21.12 ± 8.80	14.24 ± 5.48	**0.029**
SFA (mg/d)	37.54 ± 11.27	27.37 ± 6.58	18.86 ± 5.14	0.385
Trans fat	0.001 ± 0.002	0.001 ± 0.002	0.001 ± 0.003	0.608
Oleic acid (g/d)	34.87 ± 9.55	29.18 ± 9.32	19.55 ± 6.46	**0.027**
Linolenic acid (g/d)	1.58 ± 0.55	1.26 ± 0.67	0.82 ± 0.40	0.073
Linoleic acid (g/d)	20.80 ± 7.42	18.44 ± 8.59	12.27 ± 5.34	**0.030**
EPA (g/d)	0.03 ± 0.04	0.03 ± 0.04	0.02 ± 0.02	0.833
DHA (g/d)	0.12 ± 0.13	0.10 ± 0.12	0.08 ± 0.08	0.948

CSI: Cholesterol to saturated fat index; DHA: docosahexaenoic acid; EPA: eicosapentaenoic acid; MUFA; monounsaturated fatty acid; PUFA: polyunsaturated fatty acid; SFA: saturated fatty acid; T: tertile; TC: total cholesterol

Data are mean ± SD

P-value*: ANCOVA was performed to adjust the potential confounding factor (energy intake)

p < 0.05 was considered significant

### Psychological disorders of study participants according to tertiles of CSI and N6/N3

The psychological disorders of study participants according to tertiles of CSI and N6/N3 were presented in Table 3. In the crude model, a marginally significant mean difference was observed among tertiles of the CSI in terms of stress (P = 0.051), DASS-21(P = 0.078), and there were no significant differences in any other variables across tertiles of CSI and N6/N3 (P > 0.05). After adjusting with confounders (age, energy intake, BMI, and physical activity) the mean differences of stress, anxiety, depression, DASS-21 across tertiles of CSI and N6/N3 were not significant (P < 0.05).

**Table 3:** Psychological disorders of study participants according to tertiles of CSI (378) and N6/N3 (279) in obese and overweight women

**Variables†**	**CSI**			**P-value**	**P-value**_**b**_
	**Mean ± SD**				
	**T**_**1**_ **(n = 126)**	**T**_**2**_ **(n = 126)**	**T**_**3**_ **(n = 126)**		
Stress	8.83 ± 5.48	7.04 ± 4.74	8.01 ± 4.54	**0.051**	0.109
Anxiety	5.28 ± 4.03	4.58 ± 3.64	5.36 ± 3.86	0.334	0.456
Depression	6.00 ± 5.09	4.62 ± 4.31	5.04 ± 4.44	0.124	0.340
DASS-21	20.11 ± 12.74	16.25 ± 10.83	18.41 ± 11.05	**0.078**	0.284
**Variables†**	**N6/N3**			**P-value**	**P-value**_**b**_
	**Mean ± SD**				
	**T**_**1**_ **(n = 93)**	**T**_**2**_ **(n = 93)**	**T**_**3**_ **(n = 93)**		
Stress	7.88 ± 4.62	7.82 ± 5.41	8.09 ± 4.96	0.935	0.897
Anxiety	4.68 ± 3.55	4.70 ± 3.82	5.76 ± 4.10	0.113	0.459
Depression	4.95 ± 4.58	4.85 ± 4.18	5.86 ± 5.14	0.293	0.842
DASS-21	17.51 ± 10.99	17.38 ± 11.45	19.72 ± 12.49	0.341	0.854

CSI: Cholesterol to saturated fat index; GRS: genetic risk scores; SD: standard deviation; T: tertile

† Calculated by analysis of variance (ANOVA)

b: Adjusted for age, BMI, physical activity, and total energy intake

a: BMI considers as a collinear variable for anthropometric measurements and these variables are adjusted for age, physical activity, and total energy intake

p < 0.05 was considered significant

### The interaction between GRS with tertiles of CSI and N6/N3 on psychological disorders

The interaction between GRS with tertiles of the CSI and N6/N3 ratio on psychological disorders were presented in Table 4. In the crude model, a positive interaction was observed between GRS and T3 of N6/N3 ratio on anxiety (β = 0.79, CI = 0.02,1.57, P = 0.044), depression (β = 1.08, CI = 0.14,2.02, P = 0.023), DASS-21 (β = 2.52, CI = 0.17,4.88, P = 0.036). After adjusting for age, energy intake, physical activity and BMI in model 1, the interaction between GRS and T3 of N6/N3 ratio on anxiety (β = 0.91, CI = 0.08,1.75, P = 0.031), depression (β = 1.05, CI = 0.06,2.04, P = 0.037), DASS-21 (β = 2.22, CI= -0.31,4.75, P = 0.086) remained positive. By contrast, GRS in the interaction with tertiles of CSI showed no significant interaction on stress, anxiety, depression, DASS21, not in the crude model, and even after adjustment in model1.

**Table 4:** The interaction between GRS with CSI (378) and N6/N3 (279) on psychological disorders in obese and overweight women

**Variable**	**GRS**	**CSI**												
		**T1**	**T2**						**T3**					
			Crude			Model 1			Crude			Model 1		
			B	CI	P	B	CI	P	B	CI	P	B	CI	P
Stress		Reference	0.51	-0.43, 1.45	0.290	0.76	-0.24, 1.77	0.137	-0.16	-1.26, 0.93	0.766	-0.11	-1.29, 1.06	0.851
Anxiety		Reference	-0.03	-0.76, 0.70	0.936	0.16	-0.60, 0.92	0.678	-0.44	-1.30, 0.40	0.305	-0.73	-1.62, 0.16	0.108
Depression		Reference	-0.13	-1.01, 0.75	0.769	0.14	-0.75, 1.05	0.750	-0.66	-1.69, 0.36	0.207	-0.76	-1.81, 0.29	0.159
Dass-21		Reference	0.34	-1.86, 2.55	0.758	1.07	-1.23, 3.38	0.361	-1.27	-3.84, 1.29	0.331	-1.60	-4.30, 1.09	0.244
**Variable**	**GRS**	**N6/N3**												
		**T1**	**T2**						**T3**					
			Crude			Model 1			Crude			Model 1		
			B	CI	P	B	CI	P	B	CI	P	B	CI	P
Stress		Reference	0.50	-0.51, 1.52	0.336	0.18	-0.92, 1.29	0.745	0.64	-0.37, 1.66	0.218	0.25	-0.86, 1.36	0.661
Anxiety		Reference	0.49	-0.28, 1.26	0.211	0.41	-0.41, 1.24	0.332	0.79	0.02, 1.57	**0.044**	0.91	0.08, 1.75	**0.031**
Depression		Reference	0.70	-0.23, 1.64	0.141	0.48	-0.50, 1.46	0.335	1.08	0.14, 2.02	**0.023**	1.05	0.06, 2.04	**0.037**
Dass-21		Reference	1.69	-0.65, 4.05	0.157	1.07	-1.44, 3.60	0.402	2.52	0.17, 4.88	**0.036**	2.22	-0.31, 4.75	**0.086**

CI: confidence interval; CSI: cholesterol to saturated fat index; GRS: genetic risk scores; T: tertile

GLM was performed to identify the interaction between GRS with CSI and N6/N3 on psychological disorders

model 1 = adjusted for potential confounding factors including (age, energy intake, physical activity and BMI)

a: BMI considered as collinear for body fat percentage and waist circumference, and these variables are adjusted for age, physical activity, and total energy intake

p < 0.1 was considered significant

## Discussion

This cross-sectional study aimed to investigate the interaction between fatty acid quality indices and GRS based on BMI-linked genetic markers, namely, MC4R (rs17782313), CAV-1(rs3807992) and Cry-1 (rs2287161) on mental health in overweight and obese women. We discovered that dietary fatty acid quality evaluated by N-6/N-3 ratio regarding genetic predisposition were predictive of mental disorder in our population.

Our findings revealed a positive significant interaction between greater GRS with high ratio of N-6 to N-3 on anxiety, depression, and Dass-21. There was no significant interaction in terms of CSI with mental variables, though. Given the lack of previous research on the fatty acid quality indices and mental health and even less reflected the relationship of the mental abnormalities with lipid profiles regarding genetic susceptibility, our results shed light on an unknown interaction between dietary fat quality measurements and genetic predisposition on mental disorders. According to a previous study, consumption of a high fat diet, compared to a normal diet, in mice led to more secretion of CAV-1 in adipose tissue [46]. Apart from that, CAV-1 expression has been shown to be involved in depression [47] and CAV-1 knockdown could reverse the development of this disorder [48]. MC4R is expressed in different parts of central nervous system, such as hypothalamus, brain stem, cerebral cortex, and spinal cord [49]. A non-significant interaction was observed between MC4R minor allele and daily increased fat intake with high stress in Korean adults [50]. Considering Cry-1 rs2287161, Soria et al. demonstrated association between this marker with the susceptibility of the Major Depressive Disorder (MDD) [51]. One publication among British participants indicated a genetic-based direct association between increased lipid metabolism abnormalities and serum total cholesterol with MDD [52]. The association of BMI together with a GRS has also been shown to predict depression and phobic anxiety [53–56]. One explanation for this association depends on the possible positive relationship between BMI-related genes and promoting obesity with intake of total dietary fat, as the least-satiating macronutrient [57, 58], and SFAs [59–61]. Similarly, it has been reported that people with a high SFA intake and elevated scores of obesity GRS had higher BMI compared with people with low SFA consumption [62]. On the other hand, in three large prospective cohorts of US participants, long-chain n–3 PUFA intakes were found to attenuate the genetic association with long-term BMI and weight changes [63].

Genetic liability for psychotic disorders is hypothesized to be related to immune dysregulation [64, 65]. The negative health outcomes of abovementioned higher BMI and obesity on brain function might be explained by inflammation processes [66]. More so, an imbalance of omega-6 and omega-3 PUFA through inflammatory cytokine overproduction have been shown to be involved in the pathology of depression [67] and have worse impact on cognition [68–71]. Notably, it has been reported that higher N-6/N-3 ratio was correlated with higher HOMA-IR and FBS as well as increased serum hs-CRP concentration [72]. We also observed consistent results in terms of HOMA-IR and FBS. Further, there is some suggestion that higher concentration of omega-3s may have neuroprotective functions through their effects on inflammation and oxidative stress as well as insulin resistance, while omega-6s might be important mediators of inflammation by producing eicosanoid products [73–76]. As such, higher N-6/N-3 ratio in individuals with elevated scores of GRS appears to have unfavorable effects on mental health and these findings focus on the need to balance the omega-6/omega-3 essential fatty acid for prevention mental illness and improve health.

To our knowledge, our study is the first to examine the joint interaction of BMI-GRS including MC4R (rs17782313), CAV-1 (rs38 07992), and Cry1 (rs2287161) and dietary fat quality indices with mental health in overweight and obese women. As an additional strength, we applied BMI-GRS rather than specific single SNPs to identify high-risk groups and predict interactions between GRS and dietary fatty acid quality with mental health. However, some potential limitations should be considered. First of all, the cross-sectional study design prevented us establishing causality. Second, the use of self-reported dietary questionnaire is prone to reporting bias. Furthermore, the current study with small sample size included only overweight and obese women precludes the generalizability of our findings to all populations.

## Conclusion

This study indicates a positive link between the ratio of N-6 to N-3 with mental health-related variables in individuals with elevated GRS, highlighting the importance of appropriate amounts of dietary omega-6 and omega-3 for management of aforementioned disorders. However, as a result of the limited literatures conducted in this area, further prospective studies in different populations will be needed to validate these findings.

## Data Availability

The datasets generated and /or analyzed during the current study are not publicly available due to preserving participant anonymity but are available from the Khadijeh Mirzaei on reasonable request.

## Abbreviations

GRS: Genetic risk score
GLM: Depression Anxiety Stress Scales
DASS-21: Generalized linear model
MDD: Major Depressive Disorder
GWAS: Genome-wide association studies
SNP: Single nucleotide polymorphism
CRY: Cryptochrome
MC4R: Melanocortin-4 Receptor
CAV: Caveolin
EFA: Essential fatty acids
BF%: Body fat percentage
BFM: Body fat mass
BMI: Body mass index
CSI: Cholestrol to saturated fat index
DBP: Diastolic blood pressure
FBS: Fasting blood sugar
FFM: Fat free mass
HDL: High density lipoprotein
HOMA: Homeostatic model assessment
hs-CRP: High-sensitivity C-reactive protein
PA: Physical activity
SD: Standard deviation
SBP: Systolic blood pressure
TC: Total cholesterol
TG: Triglyceride
VFA: Visceral fat area
WC: Waist circumference
